# Association of the *BDNF* rs6265 Polymorphism with Cognitive Impairment in Multiple Sclerosis: A Case–Control Study in Mexican Patients

**DOI:** 10.3390/genes14122130

**Published:** 2023-11-26

**Authors:** Adriana Aguayo-Arelis, Brenda Viridiana Rabago-Barajas, Ana Miriam Saldaña-Cruz, Miguel Ángel Macías-Islas

**Affiliations:** 1 Departamento de Psicología Aplicada, Centro Universitario de Ciencias de la Salud, Universidad de Guadalajara, Ameca 46600, Mexico; aaguayo25@gmail.com (A.A.-A.); brenda.rabago@gmail.com (B.V.R.-B.); 2 Instituto de Terapéutica Experimental y Clínica, Departamento de Fisiología, Centro Universitario de Ciencias de la Salud, Universidad de Guadalajara, Ameca 46600, Mexico; ana.saldanac@academicos.udg.mx

**Keywords:** BDNF, cognitive impairment, multiple sclerosis, polymorphism

## Abstract

Cognition is a set of brain processes that allow the individual to interact with their environment. Multiple sclerosis (MS) is a chronic inflammatory disease that affects the cerebral white matter of the brain cortex and spinal cord, leading to cognitive impairment (CI) in 40–60% of the patients. Many studies have determined that CI is linked to genetic risk factors. We aimed to evaluate the association between *BDNF* gene rs6265 polymorphism and cognitive impairment in Mexican patients with MS by performing a case–control study. Mestizo-Mexican patients diagnosed with MS based on McDonald’s criteria were enrolled. Cases were MS patients with CI (*n* = 31) while controls were MS patients without CI (*n* = 31). To measure cognitive functioning in MS patients, a neuropsychological screening battery for MS (NSB-MS) was used. Genotyping of the rs6265 gene variant was performed using quantitative real-time PCR (qPCR) with TaqMan probes. The results showed no statistically significant differences in sociodemographic and disease variables between case and control groups. qPCR analysis showed that there were 68% Val/Val wild-type homozygotes, 29% Val/Met polymorphic heterozygotes, and 3% Met/Met polymorphic homozygotes. The presence of *BDNF* gene rs6265 polymorphism showed an increased probability (3.6 times) of global cognitive impairment.

## 1. Introduction

Multiple sclerosis (MS) is the most common and disabling neurological disease in young adults. This condition affects more than 2.5 million people around the world. Because of the great variability in the symptoms and the unpredictable clinical course, the diagnosis of MS and treatment indications are difficult to establish [1].

Although earlier evidence showed an inflammatory demyelinating phenotype limited to the brain white matter, it is now known that it affects the cerebral cortex, permanently damages the axons of neurons, and causes neurodegenerative changes from the early period of their evolution. Its origin may also be associated with a combination of predisposing genetic factors, demonstrating a homozygous twin concordance of 30%. Additionally, biological vectors such as viruses, with Epstein–Barr virus being the most frequent, have been associated with this condition. Failures in immune recognition mechanisms have also been proposed [2].

The clinical course of MS has an initial phase of inflammatory predominance that progresses to neurodegenerative mechanisms. Current disease-modifying therapies (DMT) have been effective against the inflammatory phase, but their impact on delaying progression is not fully demonstrated [3].

Along with motor symptoms associated with MS, cognitive impairment (CI) is another symptom that impacts the health-related quality of life. This condition occurs in 40–60% of cases despite the clinical course and disease evolution. Usually, CI is more evident in the advanced stages of the disease. Several cognitive processes can be affected but the most frequent include decreased information processing speed and impairment of visual and verbal memory. Despite MacDonald’s diagnosis criteria, magnetic resonance imaging (MRI) and brain volumetry analysis are typically used to measure neurodegeneration and cognitive impairment; this earlier evidence should be confirmed using neuropsychological evaluation instruments. Although there is information on the frequency of neuropsychological factors in MS, factors associated with the appearance of CI, as well as factors that likely prevent them, are still unknown [4,5].

Currently, there are no validated and definitive biological markers for the diagnosis of MS and the associated CI. However, some cytokines have been associated with these pathologies, including TNF-α, CXCL8, IL-15, IL-12p40, and CXCL13 [6]. Other factors have been associated with CI in MS, including age, gender, time of disease course, physical disability status, brain atrophy, genetic predisposition, and some factors associated with brain plasticity. Brain-derived neurotrophic factor (BDNF) is a protein encoded by a gene with the same name that regulates developmental processes in the nervous system including cell survival, growth, differentiation, and neuronal plasticity. In fact, there is increasing research interest in studying this neurotrophin as a promising molecule that plays a neuroprotective role in MS [7].

Polymorphisms are molecular markers responsible for the variability between individuals of the same species. They have multiple applications and are used in medicine to detect individual susceptibility to developing a health condition or differences in treatment outcomes. The rs6265 polymorphism in the *BDNF* gene is located on the short arm of chromosome 11 in band 14.1. It is a single base mutation that changes guanine to adenine at position 196 (G196A) and causes an amino acid substitution from methionine to valine. Some molecular studies have shown that the presence of the Met variant is sufficient to induce a low production of BDNF [8]. These modifications lead to structural damage and retraction in the growth of new neurons [9]. In mature neurons, it causes depletion of dendrites and inhibits long-term potentiation, thus causing alterations in communication and brain plasticity [10,11].

Interestingly, current research has shown that the *BDNF* gene rs6265 polymorphism is a genetic variant that is sufficient to produce low BDNF copies, which promotes structural changes in neurons and impairs brain plasticity [12,13]. Our study focuses on the brain plasticity associated with BDNF, since when an individual suffers an alteration that can compromise the normal functioning of the brain, different brain plasticity factors begin to act to minimize the damage [14]. The objective of this case–control study was to analyze the association between *BDNF* gene rs6265 polymorphism and CI in an ethnically homogeneous cohort of MS patients from Western Mexico.

## 2. Materials and Methods

### 2.1. Study Design

A case–control study was performed in a cohort of relapsing-remitting MS (RRMS) with CI (cases) or without CI (controls). The protocol was approved by the local research ethics committee of the Instituto de Terapéutica Experimental y Clínica of the University of Guadalajara (approval number: CEI/485/2019). This study was carried out in the facilities of the Unidad de Atención en Neurociencias from UDG. All participants were enrolled and treated at the Mexican Foundation for Multiple Sclerosis, A.C. (Guadalajara, Jalisco, Mexico) from 24 January 2019 to 31 August 2021.

### 2.2. Study Population

All individuals were aged ≥18 years and were Mexican-Mestizo as defined by the National Institute of Anthropology and History (INAH). Mexican-Mestizos have been defined by INAH as “individuals born in Mexico from the original autochthonous inhabitants of the region and mainly Spaniards” [15].

**Figure 1:**
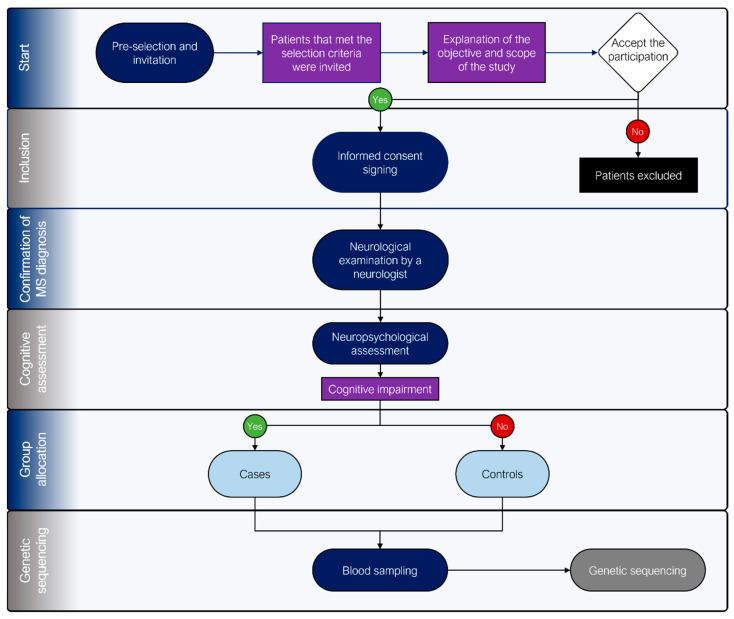
Flowchart of the selection, group allocation, and genotyping processes in the study.

A group of MS patients from the Mexican Foundation for Multiple Sclerosis, A.C. who met the selection criteria were invited to participate. Those who agreed and signed an informed consent form were physically and neurological examined by an expert neurologist to confirm diagnosis. Once confirmed, a set of neuropsychological tests was applied to explore the presence or absence of CI. A non-randomized group allocation process was performed to generate both cases (CI detected) and control (no CI detected) groups. Finally, a blood sample was taken from all patients to perform genotypification (Figure 1).

### 2.3. Sample Size

The sample size was calculated using the formula in the Epi Info™ statistical package version 7.0 (Centers for Disease Control and Prevention, Atlanta, GA, USA) to compare two proportions. We calculated the sample size based on a frequency of 40% cognitive damage in patients with MS, a confidence level of 95% (α = 0.05), and a statistical power of 80%. This calculation resulted in 31 patients per group.

### 2.4. Physical and Neurological Examinations

Patients diagnosed with RRMS were included and re-assessed by an experienced neurologist based on their early clinical history and a new physical examination. A full neurological examination allowed us to ensure that the patients fulfilled the 2017 McDonald clinical criteria. We excluded patients with severe visual or auditory impairment, substance abuse, relapses in the last 30 days, and other uncontrolled autoimmune or psychiatric diseases.

### 2.5. Neuropsychological Instruments

To measure the cognitive functioning of MS patients, a neuropsychological screening battery for MS (NSB-MS) was used. This battery is a clinical and research tool useful in identifying cognitive impairments in MS patients. It comprises 377 items and 5 subtests. The sensitivity and specificity values are 71% and 94%, respectively. The application time varied between 25 and 35 min. Once the test results are obtained, the evaluator can determine the cognitive status of MS patients [16,17].

In this context, six tests comprise the NSB-MS. Briefly, the selective memory test measures short-term storage (STM) and recovering long-term (LTM) memory; the 7/24 spatial recall test (7/24 SR test) measures visual memory; the paced auditory serial addition test (PASAT) measures the speed of information processing, working memory, and executive skills; the symbol digit modalities test (SDMT) measures attention; and finally, the verbal fluency (VF) test is useful for measuring thinking and language skills [18,19,20,21,22].

Control and case groups were stratified as follows: A case was any MS patient with 2 or more test scores below 1.5 standard deviations. The control group comprised patients with MS whose test results were average (+/− 1.5 standard deviations) at the time of the neuropsychological evaluation [4,23,24].

### 2.6. Quantitative Real-Time PCR

DNA extraction was performed from a 5 mL EDTA blood sample using the modified Miller method [25]. Briefly, DNA samples were placed in 1.5 µL propylene microtubes (labeled and sealed) containing TE buffer (50 µL) and frozen at −80 °C. Concentration and purity were determined using a 2000/2000 c NanoDrop™ device (Thermo Fischer Scientific^®^, Waltham, MA, USA). Subsequently, 20 ng/µL dilutions were made using TE buffer and placed in 200 µL propylene microtubes (Eppendorf™, Hamburg, Germany) to form the working samples. Once the extracted DNA had reached optimal conditions, it was frozen at −80 °C for polymorphism identification.

*BDNF* rs6265 polymorphisms were determined by real-time polymerase chain reaction (RT-PCR) using an allelic discrimination technique based on TaqMan^®^ probes following protocol ID: C__11592758_10. A StepOne^®^ RT-PCR kit (Applied Biosystems^®^, Foster City, CA, USA) was used for this methodology. RT-PCR cycles were as follows: denaturation (initiation at 95 °C for 10 min) followed by 40 cycles of denaturation at 95 °C for 15 s and extension at 60 °C for 60 s. Genotypification of the DNA samples was performed in duplicate. The presence of wild-type and polymorphic genotypes was determined by comparing the relative fluorescence endpoints.

### 2.7. Statistical Analysis

Qualitative variables were expressed as frequencies (%) while quantitative variables were reported as mean ± standard deviation (SD). Genotype frequencies were identified by direct counting. Allele frequencies were determined by counting from the observed genotype frequencies. Statistical comparisons of variables were performed using a Chi-square test (Fisher exact test if required). The odds ratios (ORs) and their 95% confidence intervals (95% CIs) were calculated. An OR analysis was performed as follows: (a) dominant model (CC vs. CT + TT) and (b) recessive model (CCv + CT vs. TT). A *p*-value was considered significant at less than or equal to 0.05. SPSS^®^ version 23.0 software (SPSS Inc., Chicago, IL, USA) was used for all statistical analysis. ORs and 95% CIs were calculated using Epi Info™ version 7.2 software (Atlanta, GA, USA).

## 3. Results

A total of 63 evaluations were performed in the study population; 31 corresponded to the cases group and 32 corresponded to the control group. The first statistical analysis was conducted to determine homogeneity between the study groups. The results indicated no statistically significant differences in sociodemographic or clinical variables (Table 1).

**Figure 2:**
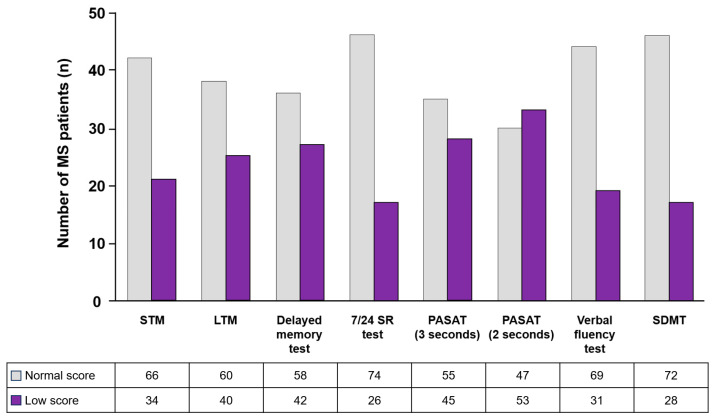
Cognitive performance of MS patients measured using Rao’s brief repeatable battery of neuropsychological tests. Light-gray bars represent normal scores and purple bars represent low scores. STM: short-term storage memory; LTM: recovering long-term memory; 7/24 SR test: 7/24 spatial recall test; PASAT: paced auditory serial addition test; SDMT: symbol digit modalities test.

Further analyses were conducted to demonstrate our primary and secondary endpoints. The cognitive performance of MS patients measured using Rao’s neuropsychological battery test showed that the most affected cognitive process was information processing speed, while attention (SDMT) and visual memory (7/24 SR test) were most conserved (Figure 2).

Genotypification results showed that 32% of MS patients had the *BDNF* r26265 polymorphism (Table 2).

No relationship was found between the presence of the polymorphism and the cognitive processes evaluated (Table 3).

The presence of the T allele increased the probability of developing CI by 3.56 times more than in MS patients without the polymorphism (Table 4).

## 4. Discussion

MS is a chronic autoimmune disease of the central nervous system. The immune system, as well as environmental and genetic factors, are involved in the pathology of MS. Among the most studied genetic factors are those associated with the increased risk of suffering from the disease, the aggressiveness of MS over the person’s lifetime once the diagnosis is made, as well as protective factors. Our population cohort is a group of patients diagnosed with MS who were divided for analysis based on the presence or absence of CI. Current evidence demonstrates that neither the progression of the disease measured using the EDSS nor the presence of relapses in the last year or the time of evolution of the disease determines the appearance of cognitive damage [3,4,26]. In this context, we analyzed the rs6265 polymorphism in the *BDNF* gene as a possible predisposing factor for cognitive damage in subjects with MS and found no influence of the above characteristics in our cohort. The baseline characteristics of the cohort showed that our groups had similar characteristics in terms of age, gender, and education, as well as clinical variables of the disease, including the years of evolution, level of disability measured using the EDSS scale, and the number of relapses in the last year, which showed us that the groups were homogeneous and therefore likely to be compared for the purposes of this research.

The main objective of our study was to evaluate the association between the rs6265 polymorphism in the *BDNF* gene and CI in patients with MS. The results showed that MS patients carrying the *BDNF* rs6265 polymorphism were 3.56 times more likely to have cognitive impairment compared with patients not carrying the polymorphism. Studies carried out on elderly subjects reported that the presence of the Met allele was associated with worse neuropsychological evaluation test performance compared with the presence of the Val allele [27]. In the case of Alzheimer’s disease patients, further investigations associated the presence of the Met allele with the acceleration of neurodegeneration and memory loss [28], while studies on Parkinson’s disease indicated that the presence of the polymorphism is associated with the development of CI [29].

In the case of MS, most of the studies that have tried to measure the relationship between the polymorphism and CI used magnetic resonance imaging and only focused on specific areas of the brain [30]. Overall, our results did not differ from those already published; that is, Met variants were associated with decreased cognitive function in neurodegenerative diseases as well as in the elderly [31]. To the best of our knowledge, our study is the first Mexican-population-based study that sought to measure the association between *BDNF* polymorphisms and CI in MS.

Regarding the determination of the presence of the polymorphism in patients with MS, the results of the genetic analysis showed that the wild homozygote was present in 68% of the subjects studied, while the heterozygote and polymorphic homozygote formed 32% of the samples. Zivadinov et al. reported that 33% of American MS patients studied had the polymorphism [30]. However, a similar study performed in an Italian population showed the presence of the polymorphism in 42% of MS patients [32]. Analysis of the frequency of this polymorphism in healthy vs. MS patients in the USA showed that the percentages of presentation were almost identical, with 33% in the MS population and 32% in the healthy population. In Italy, the percentages of presentation were 42% in the MS population and 51% in the healthy population. We could not perform this comparison in the Mexican population since there are no reports on it. Our results did not differ from those already reported. This could be associated with our own genomic diversity due to the effects of miscegenation [33]. Despite differences in MS prevalence across ethnic groups and genetic factors, which could affect the disease course and evolution, few studies have sought to associate disease courses with CI.

On the other hand, the presence and evolution of CI in MS patients were studied many years ago. The association between sociodemographic variables (age, education, and sex) and disease variables (time of evolution, disability status, number of relapses, and number of MRI lesions) remains uncertain and controversial [34]. To determine the cognitive performance of MS patients, we used Rao’s neuropsychological battery of tests, a set of neuropsychological tests with higher sensitivity and specificity for detecting CI [16]. The results showed that the most affected cognitive processes were information processing speed and verbal memory, while the least affected cognitive processes were visual memory and attention. Interestingly, Rao et al. previously described a marked increase in CI in MS patients [16]. However, further studies established that the most impaired neurological processes were verbal memory and attention, as well as speed of information processing and executive functioning [35,36].

Finally, we measured the association of the presence of the polymorphism with each of the cognitive processes evaluated. We did not find this association in our study. Other studies reported different results. For instance, Zivadinov et al. reported that patients carrying the Met variant showed alterations in tests of verbal memory, speed of information processing, and attention [30]. On the other hand, a recent study showed no significant differences between CI and no CI with the polymorphism. Thus, Val variant carriers showed greater brain responses to symbol tests and memory recovery, while Met carriers had increased brain connectivity between the hippocampus and cingulate cortex during memory recall tests [37]. Notably, MRI studies have reported associations with cognitive processes [38,39]. In addition to the different investigations, we also observed that the same neuropsychological tests were not used to measure the same cognitive processes, and when the same tests were used, the scoring varied. For instance, we used mean ± standard deviation to measure changes in cognitive processes, while other studies typically use Z values. A consensus in the evaluation and interpretation of neuropsychological tests is a basic need to compare evidence between studies. A country-specific scale should also be considered.

## 5. Conclusions

To the best of our knowledge, our study is the first to report the presence of the rs6265 polymorphism in the *BDNF* gene in a Mestizo population, specifically in a Mexican population with MS. The presence of the polymorphism did not differ from those already reported in American and European populations. This leads us to believe that miscegenation is a factor that generates susceptibility to various pathologies, including CI in MS.

The presence of the CT heterozygote and the TT polymorphic homozygote presented a higher risk (3.56 times) for CI in MS. The wild-type CC homozygote can be considered a protective factor for CI in Mexican MS patients.

## 6. Limitations

Our study has several limitations, one of which is the design itself since a case–control study does not allow us to assess the evolution of CI with respect to the presence or absence of polymorphisms. Second, the research was based solely on a regional sample of the Mexican population that corresponds to the western part of the country, thus the results may not be completely representative of the total population with MS in Mexico. Another probable limitation is that we did not analyze the polymorphism in a healthy population, especially considering that there are no publications on the matter to date. Regarding the measurement of cognitive function, we could have used magnetic resonance imaging in addition to other neuropsychological tests that would have allowed us to measure cognitive functioning in the patients in greater depth. We propose conducting future studies on the previously identified points to obtain more and better results on CI in MS.

## 7. Strengths

We had a sensitive and specific neuropsychological battery of tests that allowed us to diagnose CI based on levels of specificity. This evaluation let us establish the relationship between the presence of the polymorphism and CI. New research scopes were opened to create genetic profiles for the treatment of MS, specifically CI.

## Figures and Tables

**Table 1:** Baseline sociodemographic and disease characteristics in MS patients.

Variable	Cases (*n* = 31)	Controls (*n* = 32)	*p*-Value
Sociodemographic features			
Age	42.10 ± 10.67	38.38 ± 8.67	0.13
Scholarship	12.55 ± 4.21	14.22 ± 3.53	0.99
Sex, *n* (%)			
Female (%)	18 (58%)	25 (78%)	0.08
Male (%)	13 (42%)	7 (22%)	
Features of the disease			
Disease course (years)	10.87 ± 6.73	9.44 ± 6.32	0.38
EDSS	3.7 ± 1.4	3.1 ± 2.0	0.17
Relapses (last year)	0.52 ± 0.72	0.31 ± 0.59	0.22

Cases: patients diagnosed with MS plus cognitive impairment; Controls: patients diagnosed with MS without cognitive impairment; EDSS: expanded disability status scale; Statistical significance: *p* ≤ 0.05.

**Table 2:** Gene polymorphisms identified in MS patients.

Genotype	Gene Variant	Frequency
Wild-type homozygous	Val/Val	CC, *n* = 43 (68%)
Heterozygous	Val/Met	CT, *n* = 18 (29%)
Homozygous polymorphism	Met/Met	TT, *n* = 2 (3%)
Allele 2*n* = 126		
C	Wild-type	104 (82.5%)
T	Polymorphism	22 (17.5%)

Val: Valine; Met: Methionine; C: Cytosine; T: Thymine. Data are shown in frequencies and percentages.

**Table 3:** Association of the presence of the polymorphism with each of the cognitive processes evaluated.

Multiple Sclerosis (*n* = 63)	Cognitive Process/Sub-Test	OR	95% CI	*p*-Value
Dominant model (CC versus CT + TT)	Verbal memory			
	STM	1.53	0.48–4.65	0.44
	LTM	1.86	0.63–5.48	0.25
	Paced memory	0.84	0.28–2.47	0.75
	Visual memory			
	7/24 SR test	1.77	0.55–5.66	0.32
	Speed of information processing			
	PASAT, 3 s.	1.03	0.35–3.00	0.95
	PASAT, 2 s.	0.86	0.30–2.51	0.79
	Language			
	Verbal fluency	1.39	0.33–4.32	0.56
	Attention			
	SDMT	1.24	0.38–4.04	0.71

CC: homozygous genotype; CT: heterozygous genotype; TT: homozygous polymorphic genotype; STM: short-term storage memory; LTM: recovering long-term memory; 7/24 SR test: 7/24 spatial recall test; PASAT: paced auditory serial addition test; SDMT: symbol digit modalities test; Statistical significance: *p* ≤ 0.05.

**Table 4:** Evaluation of the rs6265 polymorphism as a CI predictor in MS patients.

Multiple Sclerosis (*n* = 63)	CI (*n* = 31)	No CI (*n* = 32)	OR	95% Confidence Interval	*p*-Value
Genotype					
CC, *n* = 43 (%)	17 (55)	26 (81)	-------	-------	-------
CT, *n* = 18 (%)	13 (42)	5 (16)	-------	-------	-------
TT, *n* = 2 (%)	1 (3)	1 (3)	-------	-------	-------
CT versus CC	-------	-------	3.97	1.19–13.19	0.01 *
TT versus CC	-------	-------	1.51	0.03–61.8	0.80
TT versus CT	-------	-------	0.40	0.009–17.9	0.60
Genetic models					
Dominate model (CC vs. CT + TT)	-------	-------	3.56	1.14–11.1	0.02 *
Recessive model (CC + CT vs. TT)	-------	-------	1.03	0.06–17.28	0.98
Allele, 2*n* = 126	2*n* = 62	2*n* = 64	-------	-------	-------
Allele C, 2*n* = 104 (%)	47 (76)	57 (89)	0.38	0.14–1.02	0.05 *
Allele T, 2*n* = 22 (%)	15 (24)	7 (11)	2.59	0.97–6.90	0.05 *

CI: cognitive impairment; CC: homozygous genotype; CT: heterozygous genotype; TT: homozygous polymorphic genotype. * Statistical significance: *p* ≤ 0.05.

## Data Availability

The data presented in this study are available on request from the corresponding author. The data are not publicly available due to ethical restrictions by the Mexican Foundation for Multiple Sclerosis, A.C.
